# Evaluation of rooting characteristics of *Ephedra* cuttings by anatomy and promising strain selection based on rooting characteristics and alkaloid content

**DOI:** 10.1007/s11418-023-01680-3

**Published:** 2023-01-21

**Authors:** Yoshitomi Kudo, Hirokazu Ando, Ai Kaneda, Honoka Ito, Kazuki Umemoto, Si-ran Ni, Masayuki Mikage, Yohei Sasaki

**Affiliations:** 1 Laboratory of Molecular Pharmacognosy, Graduate School of Medical Sciences, Kanazawa University, Kakuma-Machi, Kanazawa, Ishikawa 920-1192 Japan; 2 Laboratory of Medicinal Plant Resources, Department of Bio-Resource Development, Faculty of Agriculture, Tokyo University of Agriculture, 1737 Funako, Atsugi, Kanagawa 243-0034 Japan

**Keywords:** *Ephedra sinica*, Cutting, Morphology, Breeding, Alkaloid, Ephedrine, Pseudoephedrine

## Abstract

The differences in rooting characteristics of cuttings prepared from *E. sinica* strains were investigated and found that cuttings prepared from strains with high rooting characteristics showed approximately 90% of the cuttings were rooted, whereas cuttings prepared from low rooting characteristics did not root. To understand the reason for this substantial difference, the anatomy of nodes was examined and found that adventitious roots were generated from the cortex and parenchyma in pith. Calculations of the correlation coefficients between the rooting rate and the value of anatomy indicated that the rooting rate was positively correlated with the parenchyma in pith in the node. On the basis of the positive correlation, it is possible to estimate the rooting characteristics of new strains without having to prepare cuttings. Next, we conducted a screening for *E. sinica* strains on the basis of total alkaloids content [ephedrine (E) + pseudoephedrine (PE)] and selected strains having no less than 0.7% total alkaloids content as defined by the Japanese Pharmacopoeia 18th edition. Strains having characteristic E or PE content were uncovered: E-rich strains had 100% E content and PE-rich strains had 99% PE content. We were able to select *E. sinica* strains on the basis of two factors: high rooting rate of cuttings and high or characteristic alkaloid content. These strains are valuable for breeding.

## Introduction

*Ephedra sinica* is an important medicinal plant and the source of Ephedra herb. Because *E. sinica* does not grow and has not been cultivated commercially in Japan, Ephedra herb is imported from China. Although a new cultivation method of *Ephedra sinica* for the production of Ephedra herb in Japan has been trying to invent [1], we have encountered several issues in breeding and seedling production, which have hindered the production of high-quality Ephedra herb. The ratio of the main bioactive compounds in *E. sinica*, ephedrine (E) and pseudoephedrine (PE) is reported to be genetically controlled [2]. E and PE have different bioactivities. Compared with PE, E has more potent asthma-relieving effect [3, 4] and blood pressure elevating effect [5]. On the other hand, PE has stronger anti-inflammatory activity than E [6]. Thus, differences in E and PE content ratio may influence the bioactivity of Ephedra herb. *E. sinica* seedlings are generally produced by pollen mating methods in China [7, 8]. The pollen mating methods that are suitable for the Japanese environment have been established [9] and used to produce *Ephedra* seedlings. However, the seedlings produced by those methods have diverse alkaloid contents. Herbal medicines should have uniform quality; they should have uniform composition, color, shape, and morphological characteristics. Therefore, it is necessary to establish an asexual reproduction method. Promising strains of *Glycyrrhiza uralensis* have been selected for breeding and propagation by asexual reproduction [10]. To establish a domestic production method for Ephedra herb, propagation by asexual reproduction should be established as well. Clone seedling, which has the same gene as and inherits the phenotype of the parent, is useful for not only breeding but also research. *E. sinica* clone seedlings produced by suckering have the same alkaloid content as the mother plant [11, 12]. We have revealed that *E. sinica* clone seedlings can be produced using cuttings and suckering [13–17]. Because cuttings can produce more clone seedlings than suckering, we sought to develop a suitable cutting method and found that the best period for preparing cuttings is from June to September [15]. However, we also uncovered marked individual differences in rooting characteristics of the cuttings; some strains showed prolific rooting whereas others showed no rooting even though the cutting conditions were the same [15]. This means that strains that exhibit prolific rooting should be selected for clone seedling production. One major setback of using cuttings is that it needs to wait approximately four months to evaluate rooting characteristics. Thus, we have developed a new marker that can be used to evaluate rooting characteristics without using cuttings. This marker is expected to contribute to efficient breeding and seedling production.

In this study, first, the differences in rooting characteristics among strains were investigated. Then, we confirmed the stability of rooting characteristics for three years and the inheritance of characteristics by new stock. Anatomy related to rooting was investigated and calculated the correlation coefficients between the value of each anatomy and the rooting characteristics. After that, we screened for strains having characteristic E and PE contents. Strains that have characteristics suitable for cuttings were selected from strains screened in terms of E and PE content.

## Materials and methods

### Differences in rooting and germination characteristics between strains

Herbal stems were collected from sixteen strains of *E. sinica* (Strain Numbers ESK01-01 to ESK18) and two strains of *E.* sp. [Ep-13 and Ep-13 × *E. sinica* (hybrid of Ep-13 and *E. sinica*)] cultivated in the Medicinal Plant Garden of the School of Pharmacy, School of Pharmaceutical Sciences, Kanazawa University (medicinal plant garden) from July 29 to August 9, 2019. Ep-13 is the hybrid of *E. likiangensis* and *E. gerardiana* [18, 19]. In addition, herbal stems were collected from seventeen strains of *E. sinica* (Strain Numbers ESS01 to ESS17) grown in a cultivation field in Shika Town, Hakui-gun, Ishikawa Prefecture from July 29 to August 9, 2019. Cuttings were prepared following the method reported in a previous study [15]. The cuttings were inserted into sand mixed with vermiculite and then incubated in a growth chamber (room temperature: 25℃, humidity: 90%, light condition: 2500 Lux, 24 h, LPH-220SP, Nippon Medical and Chemical Instruments Co., Ltd.). Water was supplied up to a height of 1 cm from the bottom of the pot. The cuttings were taken out of the pot from November 29 to December 1, 2019, and their growth conditions were evaluated. The surviving cuttings were classified into four stages (+ + , +  − , −  + , − −) according to the rooting and germination conditions. Rooting means root extension from the base of cuttings. Germination means that buds grew from any node of the cuttings. +  + is a cutting that was rooting and germination. + − is a cutting that was rooting but not germination. −  + is a cutting that was not rooting but was germination. −  − is a cutting that survived without withering, although neither rooting nor germination was observed. Each value is shown in a percentage calculated using the number of all the cuttings. In January 2019, 80 g of Magamp K (N: P: K: Mg = 6%: 40%: 6%: 15%, HYPONeX Japan Corp., Ltd.) was added to each of the strains cultivated in the medicinal plant garden.

### Stability of rooting and germination characteristics over three years

Herbal stems were collected from two strains of *E. sinica* (Strain Numbers ESK01-01 and ESK02) cultivated in the medicinal plant garden and cuttings were prepared on July 13, 2017, July 4, 2018, and July 29 and 30, 2019. Cutting preparation and incubation methods were the same as that of the first experiment. Rooting and germination characteristics were evaluated on November 15, 2017, December 10, 2018, and November 29, 2019 in the same manner as the first experiment. In January 2019, 80 g of Magamp K (N: P: K: Mg = 6%: 40%: 6%: 15%, HYPONeX Japan Corp., Ltd.) was added to each strain.

### Inheritance of rooting and germination characteristics

Herbal stems were collected from two strains of *E. sinica* (Strain Numbers ESK01-01 and ESK15-01) and the clone seedlings of those strains (Strain Numbers ESK01-02 and ESK15-02) produced from cuttings cultivated in the medicinal plant garden, and cuttings were prepared on July 29 and 30, 2019. Cutting preparation and incubation methods were the same as that of the first experiment. Rooting and germination characteristics were evaluated on November 29, 2019 in the same manner as that of the first experiment. In January 2019, 80 g of Magamp K (N: P: K: Mg = 6%: 40%: 6%: 15%, HYPONeX Japan Corp., Ltd.) was added to each strain. Strains cultivated in Shika Town, Hakui-gun, Ishikawa Prefecture were fertilized with 12 g per strain of Sunurea (N: P: K: Mg = 40%: 0%: 0%, SunAgro Co., Ltd.) in June 2019.

### Identification of rooting part by anatomical study

The method used for anatomical study is based on our previous study [20].

Herbal stems of Ep-13 × *E. sinica* were collected on September 24, 2020. On the basis of previous report [16], cuttings were prepared by horizontal cutting. A horizontal cut was made immediately below the node of basal herbal stem, and the upper part was cut to a length of approximately 10 cm, leaving three nodes above it. The cutting incubation method was the same as that used in the first experiment. On November 2, 2020, the cuttings were dug up, excess soil was removed by washing with water, and the 2 cm base was soaked in FAA (Formalin-Acetic acid-Alcohol). After fixation, transverse and longitudinal slices of the cutting base were prepared with Plant Microtome (MTH-1, Nippon Medical & Chemical Instruments Co., Ltd.) and double-stained with 1.25% safranine (safranine O undiluted solution, Lot No. 200612, Muto Pure Chemicals Co., Ltd.) and Delafield's hematoxylin (Lot No. 200612, Muto Pure Chemicals Co., Ltd.). The stained slices were observed under a digital microscope (GLB-B1500MBITaN, Shimadzu Rika Corporation) and Moticam X3 (Shimadzu Rika Corporation), and images were analyzed using Motic Images Plus 2.3 s (Shimadzu Rika Corporation). For comparison, the longitudinal slices of herbal stems not collected for cuttings were observed in the same manner as that for Ep-13 × *E. sinica*.

### Relationship between anatomy and rooting

**Fig. 1:**
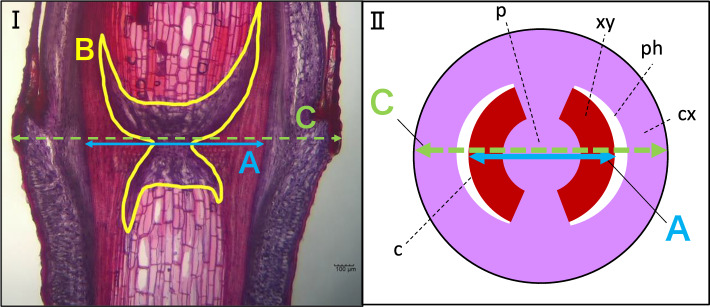
**I** Longitudinal slices of node that includes the center, and calculated part for anatomical evaluation (Strain Number ESK01). **A** The maximum distance between two cambiums. **B** Area of parenchyma in pith. **C** Diameter of herbal stem node, **II** the scheme of the transverse slice. *p* pith, *ph* phloem, *xy* xylem, *cx* cortex, *c* cambium

One part of each herbal stem from each strain that was collected for use in the experiment to determine the differences in rooting and germination characteristics among strains was kept in a − 20℃ freezer. The herbal stems of 18 strains, 20 plants (ESK01-01, ESK01-02, ESK02, ESK03, ESK07, ESK08, ESK12, ESK13, ESK15-01, ESK15-02, ESK16, ESS01, ESS06, ESS07, ESS12, ESS13, ESS14, ESS17, Ep-13, Ep-13 × *E. sinica*) were taken out from the freezer. The nodes were sampled from two or three herbal stems of each plant and fixed in FAA on October 4, 2020. Longitudinal slices of the nodes were prepared, stained, and observed in the same way as that in the first experiment. Finally, the length and area of each location (A, B, C) shown in Fig. 1I were calculated. The scheme of the transverse slice, which is shown in Fig. 1II, was created based on previous reports [21, 22]. To determine the anatomical information of each strain, two or three slices were made from different stems and averaged. Correlation coefficients between the value of each anatomy and the rooting rate, germination rate, and survival rate were calculated. The tissue of A indicates the length between the left and right cambium. The tissue of B represents the area of parenchyma in pith. The tissue of C represents the diameter of node of the herbal stem. The rooting rate is the sum of +  + and + −, the germination rate is the sum of +  + and −  + , and the survival rate is the sum of +  + , + −, −  + , and −  − .

### Selection of promising strains

#### Plant materials

Herbal stems of approximately 3,400 plants were sampled in October or November of 2015 to 2019. All plants were grown for more than three years in the field. Ephedrine (E) and pseudoephedrine (PE) contents in each plant were quantified by HPLC. Total alkaloid content (E + PE) and the content ratio of each alkaloid were calculated from E and PE contents. Using these values, plants having a high alkaloid content, or a characteristic content ratio were selected as promising strain candidates.

### Quantification of ephedrine and pseudoephedrine

#### Extraction

Sample extraction methods and conditions are described below. The methods were derived from our previous study [1]. Herbal stems of dried *E. sinica* were ground, and the obtained powder was dried in an oven at 105℃ for 15 h. To an accurately weighed pulverized sample (0.03 g) was added 1.5 mL of the extraction solution, which is the same solution as the mobile phase, and the mixture was subjected to ultrasonication for 30 min at room temperature. The resulting extract was centrifuged for 10 min at 13,000 rpm and the supernatant was passed through a membrane filter (FILTSTAR 0.45 μm, Nippon Genetics) to obtain the sample solution for HPLC.

#### HPLC conditions

HPLC analysis was performed using a Hitachi L-2200 high-performance liquid chromatograph and a COSMOSIL Packed Column 5C18-MS-II (4.6 mm I.D. × 150 mm) under the following conditions: mobile phase, CH_3_CN/H_2_O/H_3_PO_4_/SDS = 195 mL/305 mL/0.8 mL/2.4 g; column temperature, 40 °C; flow rate, 1.0 mL/min; UV detection, 210 nm; and injection volume, 10 μL. Quantification was accomplished using the absolute calibration curve method, and calibration curves were prepared using authentic ephedrine hydrochloride and pseudoephedrine hydrochloride.

#### Screening by rooting characteristics

Herbal stems were collected from 41 strains of *E. sinica* (Strain Numbers ESS18 to ESS58) cultivated in the medicinal plant garden and cuttings were prepared on July 11 and 27 and August 23, 2020. Cutting preparation and incubation methods were the same as that of the first experiment. Rooting and germination characteristics were evaluated on November 28 or December 23, 2020 in the same manner as the first experiment.

### Statistical analysis

IBM SPSS Statistics Version 25 (IBM, USA) was used for statistical analysis.

## Results

### Difference in rooting and germination characteristics between strains

**Fig. 2:**
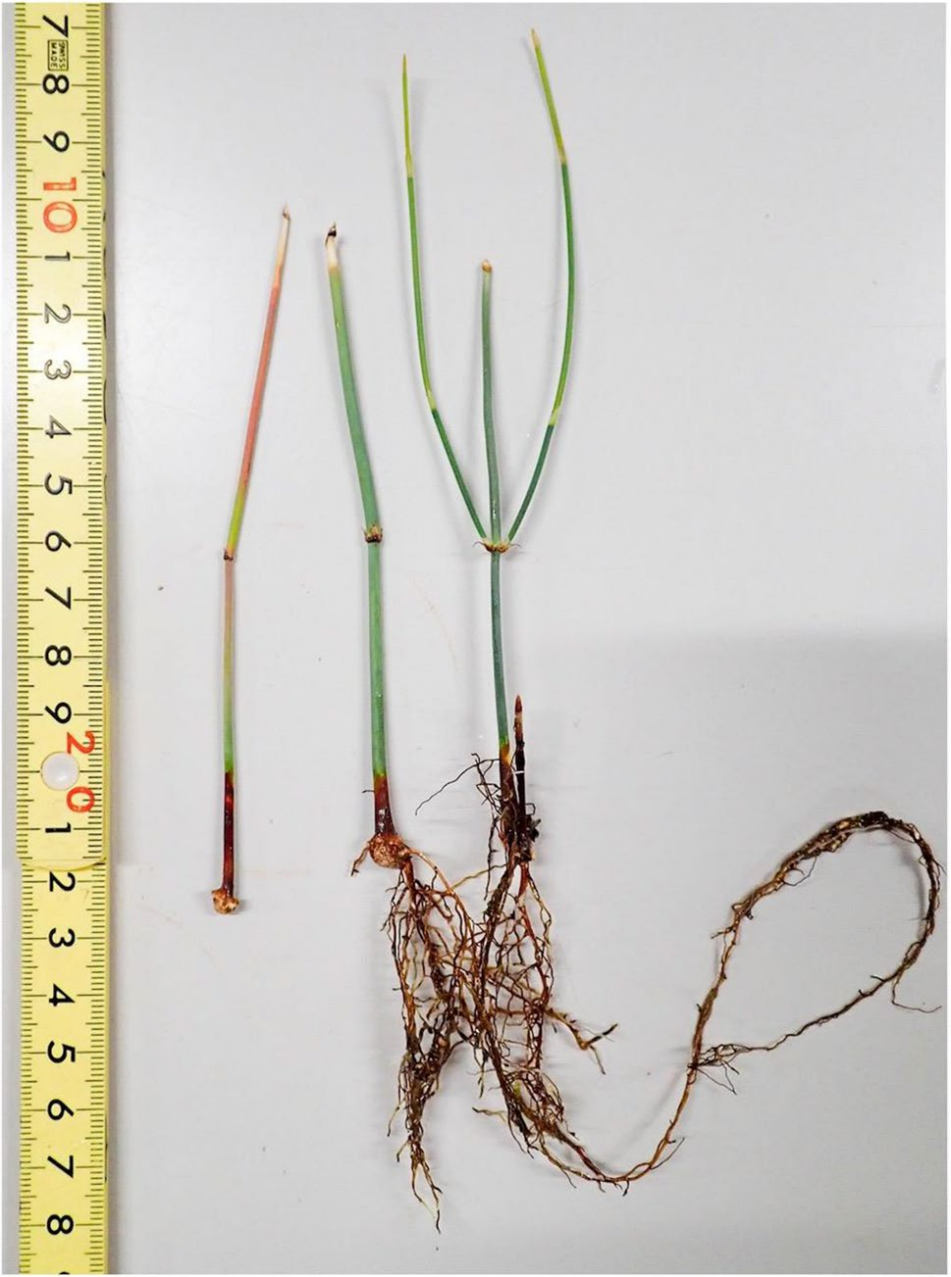
Example of seedlings produced with cuttings (Strain Number ESS06). The left cutting survived without withering, although neither roots nor sprouts were formed (− −). The middle cutting formed roots but not sprouts (+ −). The right cutting formed roots and sprouts (+ +)

**Fig. 3:**
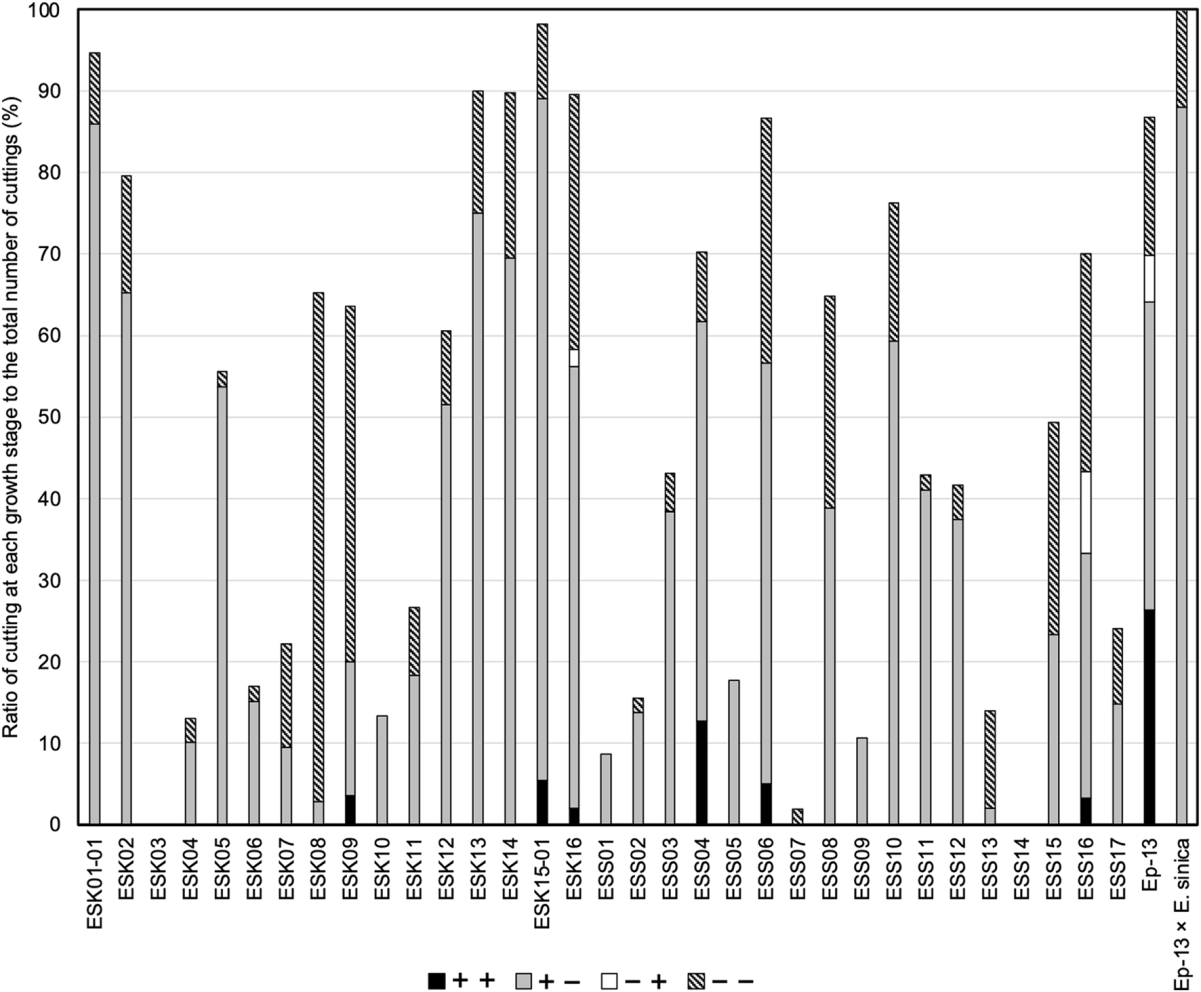
Differences in ratios of cuttings among strains. +  + : cutting that formed roots and sprouts. + −: cutting that formed roots but not sprouts. −  + : cutting that formed sprouts but not roots. −  − : cutting that survived without withering, although neither roots nor sprouts were formed

**Fig. 4:**
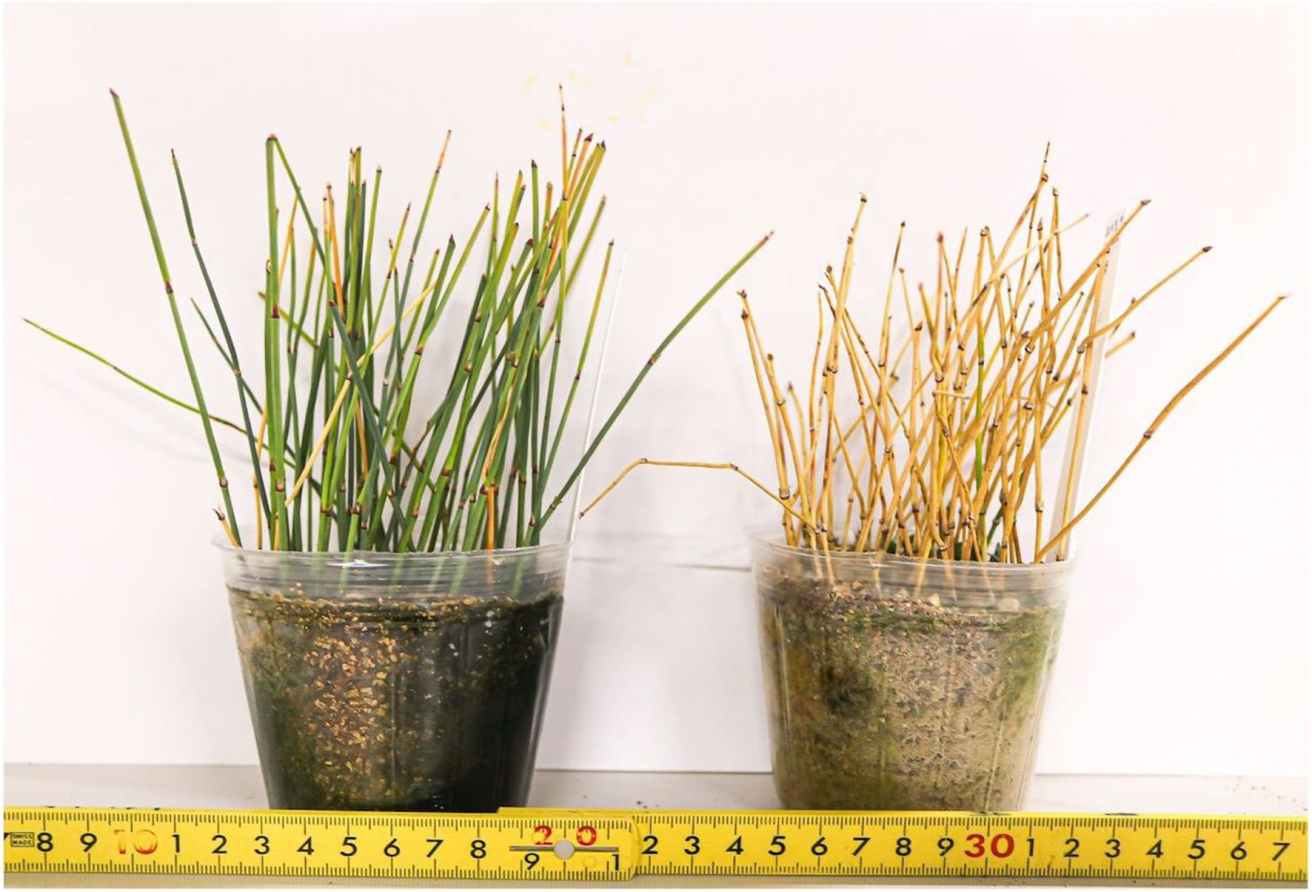
Differences in rooting characteristics among strains. Most of the cuttings in the left pot survived (Strain Number ESK02). In contrast, all of the cuttings in the right pot withered (Strain Number ESS14)

Figure 2 shows an example of the seedlings produced in this experiment. Cuttings that survived until the evaluation period varied in the condition of rooting and germination among cuttings. The rooting and germination characteristics of a total of 35 *Ephedra* strains (33 *E. sinica* strains, Ep-13, and Ep-13 × *E. sinica*) are shown in Fig. 3. Among the 33 *E. sinica* strains, ESS04 had the highest ratio of +  + cuttings (12.8%), followed by ESK15-01 (5.5%) and ESS06 (5.0%). On the other hand, 3 strains showed neither rooting nor germination. Among the 33 *E. sinica* strains, ESK01-01 had the highest ratio of + − cuttings (86.0%), followed by ESK15-01 (83.6%) and ESK13 (75.0%). The ratio of + − cuttings was 0% for three strains, 2% for ESS13, and 2.9% for ESK08. Among the 33 *E. sinica* strains, ESS16 had the highest ratio of −  + cuttings (10.0%), followed by ESK16 (2.1%), and 31 strains had no −  + cuttings. Among the 33 *E. sinica* strains, ESK08 had the highest ratio of −  − cuttings (62.3%), followed by ESK09 (43.6%) and ESK16 (31.3%). On the other hand, the ratio of −  − cuttings was 0% in six strains. The rooting characteristics for each strain differed markedly. Whereas some strains did not root at all, others showed prolific rooting (Fig. 4).

The ratio of +  + , + −, −  + and −  − in Ep-13 is 26,4%, 37.7%, 5.7% and 17.0%, respectively. The ratio of +  + , + −, −  + and −  − in Ep-13 × *E. sinica* is 0,0%, 88.0%, 0.0% and 12.0%, respectively.

### Stability of rooting and germination characteristics over three years

The rooting and germination characteristics of cuttings prepared from two *E. sinica* strains (Strain Numbers ESK01-01 and ESK02) in 2017, 2018, and 2019 are shown in Table 1. First, we explain the results for ESK01-01. In 2017, none of the cuttings were +  + or −  + , 59.0% were + −, and 30.1% were −  − ; rooting rate was 59.0%, survival rate was 89.2%, and germination rate was 0.0%. In 2018, none of the cuttings were +  + or −  + , 39.5% were + −, and 18.5% were −  − ; rooting rate was 39.5%, survival rate was 58.0%, and germination rate was 0.0%. In 2019, none of the cuttings were +  + or −  + , 86.0% were + −, and 8.8% were −  − ; rooting rate was 86.0%, survival rate was 94.7%, and germination rate was 0.0%. Next, we explain the results for ESK02. In 2017, 8.5% of the cuttings were +  + , 30.5% were + −, 10.2% were −  + , and 13.6% were −  − ; rooting rate was 39.0%, survival rate was 62.7%, and germination rate was 18.6%. In 2018, 4.3% of the cuttings were +  + , 28.3% were + −, 4.3% were −  + , and 32.6% were −  − ; rooting rate was 32.6%, survival rate was 69.6%, and germination rate was 8.7%. In 2019, none of the cuttings were +  + or −  + , 65.3% were + −, and 14.3% were −  − ; rooting rate was 65.3%, survival rate was 79.6%, and germination rate was 0.0%. The rooting rates of ESK01-01 were in the range of 39.5–86.0%, and those of ESK02 were in the range of 32.6–65.3%, showing high values over the three-year period. The rooting rate for 2017 and 2018 was relatively similar in both strains compared to 2019. On the other hand, the two strains showed high rooting rate in 2019, the year when fertilizer was applied, compared with 2017 and 2018.

**Table 1:** Stability of rooting and germination characteristics over a three-year period and inheritance of rooting and germination characteristics

	Year	+ +	+ −	− +	− −	Rooting rate	Survival rate	Germination rate
ESK01-01	2017	0.0	59.0	0.0	30.1	59.0	89.2	0.0
	2018	0.0	39.5	0.0	18.5	39.5	58.0	0.0
	2019	0.0	86.0	0.0	8.8	86.0	94.7	0.0
ESK02	2017	8.5	30.5	10.2	13.6	39.0	62.7	18.6
	2018	4.3	28.3	4.3	32.6	32.6	69.6	8.7
	2019	0.0	65.3	0.0	14.3	65.3	79.6	0.0
ESK01-02	2019	9.4	56.6	1.9	7.5	66.0	75.5	11.3
ESK15-01	2019	5.5	83.6	0.0	9.1	89.1	98.2	5.5
ESK15-02	2019	11.1	48.3	0.0	1.9	59.4	61.3	11.1

+  + : cutting that formed roots and sprouts

+ −: cutting that formed roots but not sprouts

−  + : cutting that formed sprouts but not roots

−  − : cutting that survived without withering, although neither roots nor sprouts were formed

Each value shows the ratio of cuttings at each growth stage to the total number of cuttings

The rooting rate is the sum of +  + and + −, the germination rate is the sum of +  + and −  + , and the survival rate is the sum of +  + , + −, −  + , and −  −

### Inheritance of rooting and germination characteristics

From July 29 to July 30, 2019, herbal stems of *E. sinica* strains (Strain Numbers ESK01-01, ESK15-01, ESK01-02, and ESK15-02) were collected and cut. The results for ESK01-01 in 2019 are listed in Table 1 and discussed in the preceding paragraph. With regard to ESK01-02, 9.4% of the cuttings were +  + , 56.6% were + −, 1.9% were −  + , and 7.5% were −  − ; rooting rate was 66.0%, survival rate was 75.5%, and germination rate was 11.3%. In the case of ESK15-01, 5.5% of the cuttings were +  + , 83.6% were + −, none were −  + , and 9.1% were −  − ; rooting rate was 89.1%, survival rate was 98.2%, and germination rate was 5.5%. As regards ESK15-02, 11.1% of the cuttings were +  + , 48.3% were + −, none were −  + , and 1.9% were −  − ; rooting rate was 59.4%, survival rate was 61.3%, and germination rate was 11.1%. In the new stock produced from a mother plant with high rooting rate, many cuttings formed roots, similarly to the high-rooting mother plant.

### Identification of rooting part by anatomical study

**Fig. 5:**
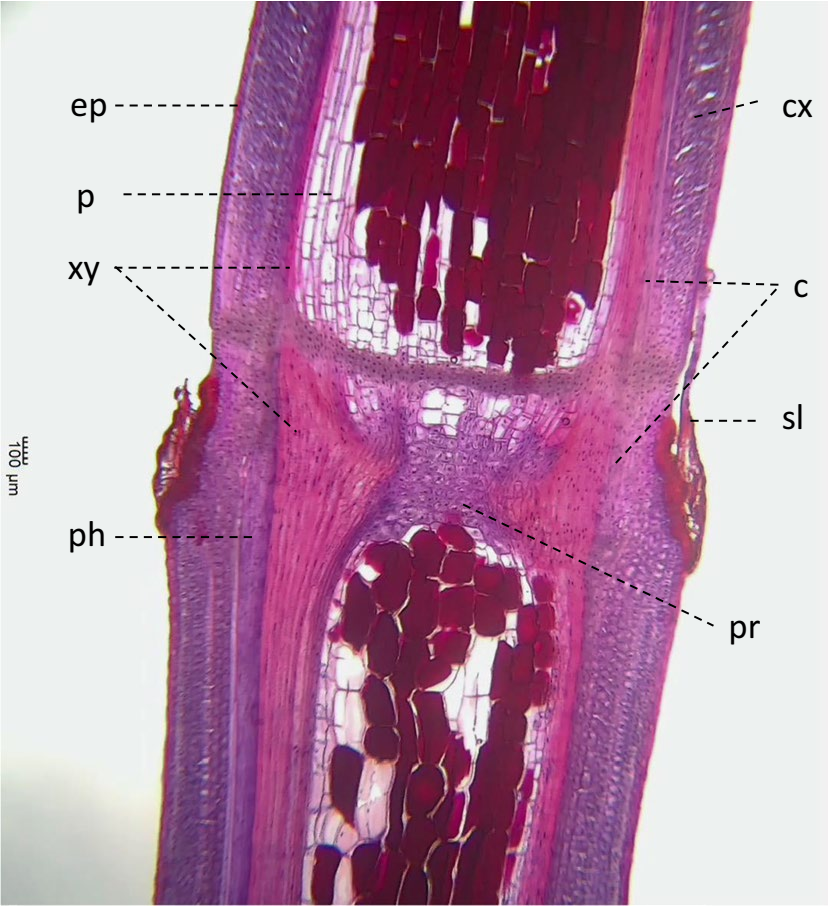
Longitudinal section of node that includes the center, cut from Ep-13 × *E. sinica* herbal stem before rooting. *p* pith, *sl* scaly leaf, *ph* phloem, *xy* xylem, *ep* epidermis, *cx* cortex, *c* cambium; *pr* parenchyma in pith

**Fig. 6:**
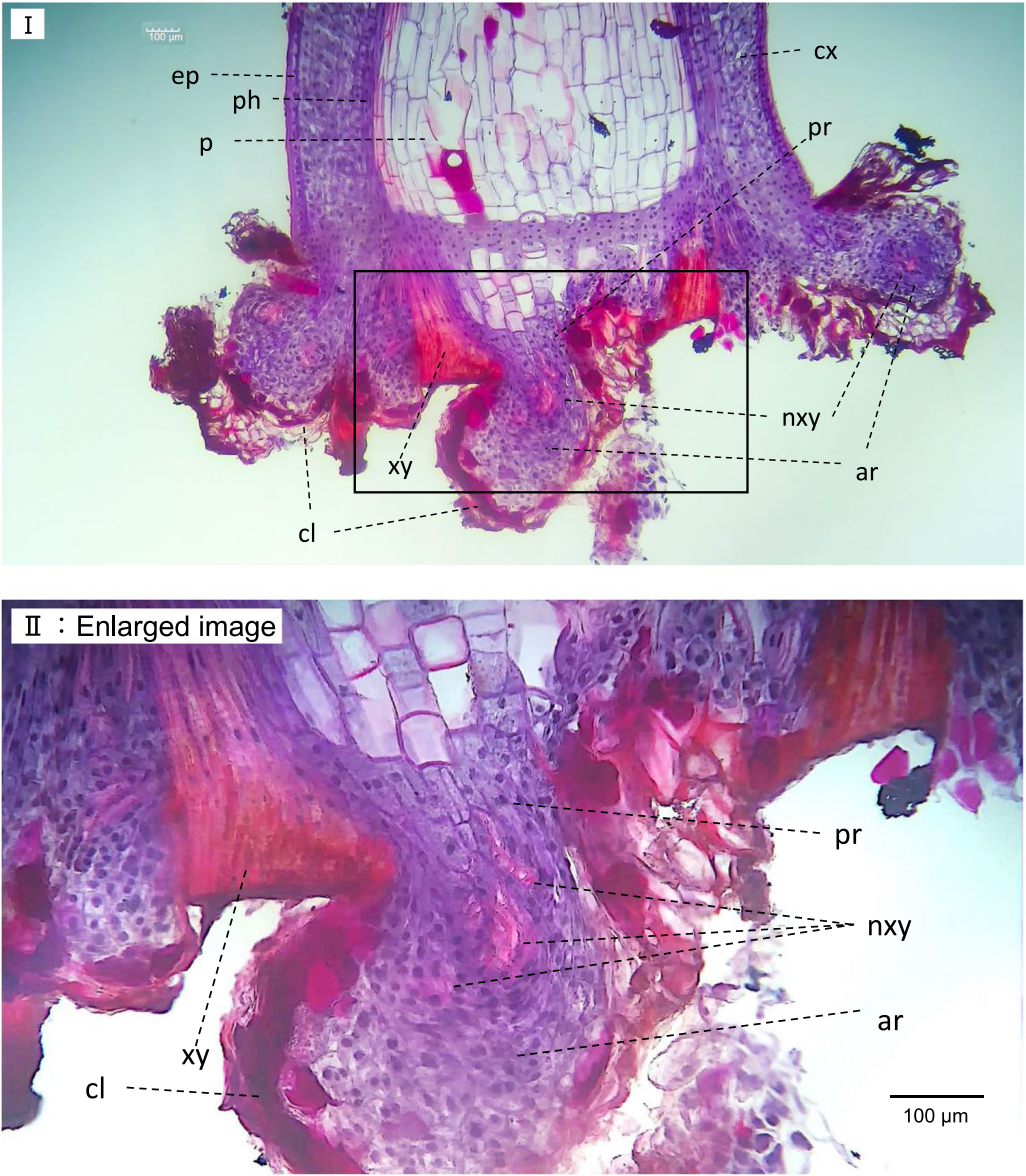
**I** Longitudinal sections of basal part of cutting after rooting. **II** Enlarged image of A. The enlarged area is shown as a square in A. The cutting was prepared from Ep-13 × *E. sinica*. *p* pith, *ph* phloem, *xy* xylem, *ep* epidermis, *cx* cortex, *nxy* new xylem, *ar* adventitious root, *cl* cork layer, *pr* parenchyma in pith

**Fig. 7:**
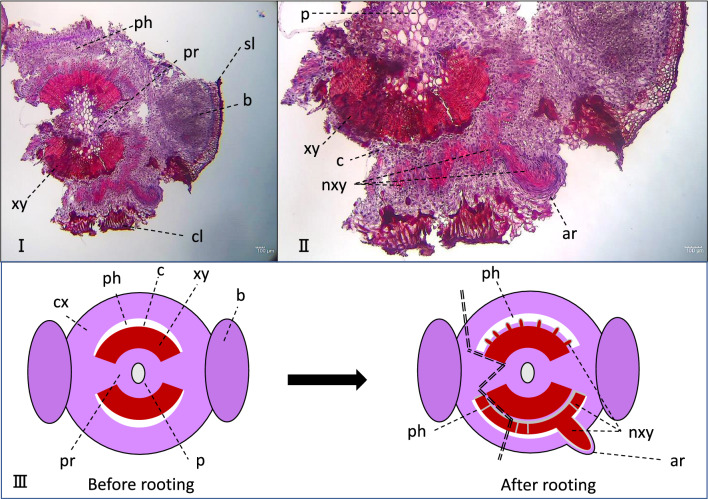
**I** Transverse sections of the node of cutting after rooting. **II** Enlarged image of A. **III** The schemes of anatomical change by rooting. After rooting, the left side of the double dashed line is the location lost by rooting in Fig. 7I and II. *p* pith, *sl* scaly leaf, *ph* phloem, *xy* xylem, *cx* cortex, *nxy* new xylem, *ar* adventitious root, *cl* cork layer, *b* bud, *c* cambium, *pr* parenchyma in pith. The cutting was prepared from Ep-13 × *E. sinica*

Figure 5 shows the longitudinal section of Ep-13 × *E. sinica* before rooting. Xylem, stained red by safranin, is more developed in the node than internode. Phloem is observed on the outside of xylem, and the middle tissue between phloem and xylem is cambium. Different from internodal anatomy parenchyma was observed in the pith in node. The longitudinal section of the basal part of cutting after rooting is shown in Fig. 6, and the transverse section of the node of cutting after rooting is shown in Fig. 7I and II. The scheme of anatomical changes caused by rooting is shown in Fig. 7III. The scheme was created based on previous studies [21, 22]. Same as the longitudinal section, parenchyma was observed in the pith in Fig. 7I and II. New xylems were developed from cambiums. After rooting, hypertrophy of the cut part and rooting from the outer cortex of the vascular bundle and the parenchyma in pith were visible. In addition, vigorous development of the cork layer was observed in the adventitious roots. In the longitudinal section, the development of new xylem and adventitious roots from the outside of the vascular bundle was apparent.

### Relationship between anatomy and rooting

**Fig. 8:**
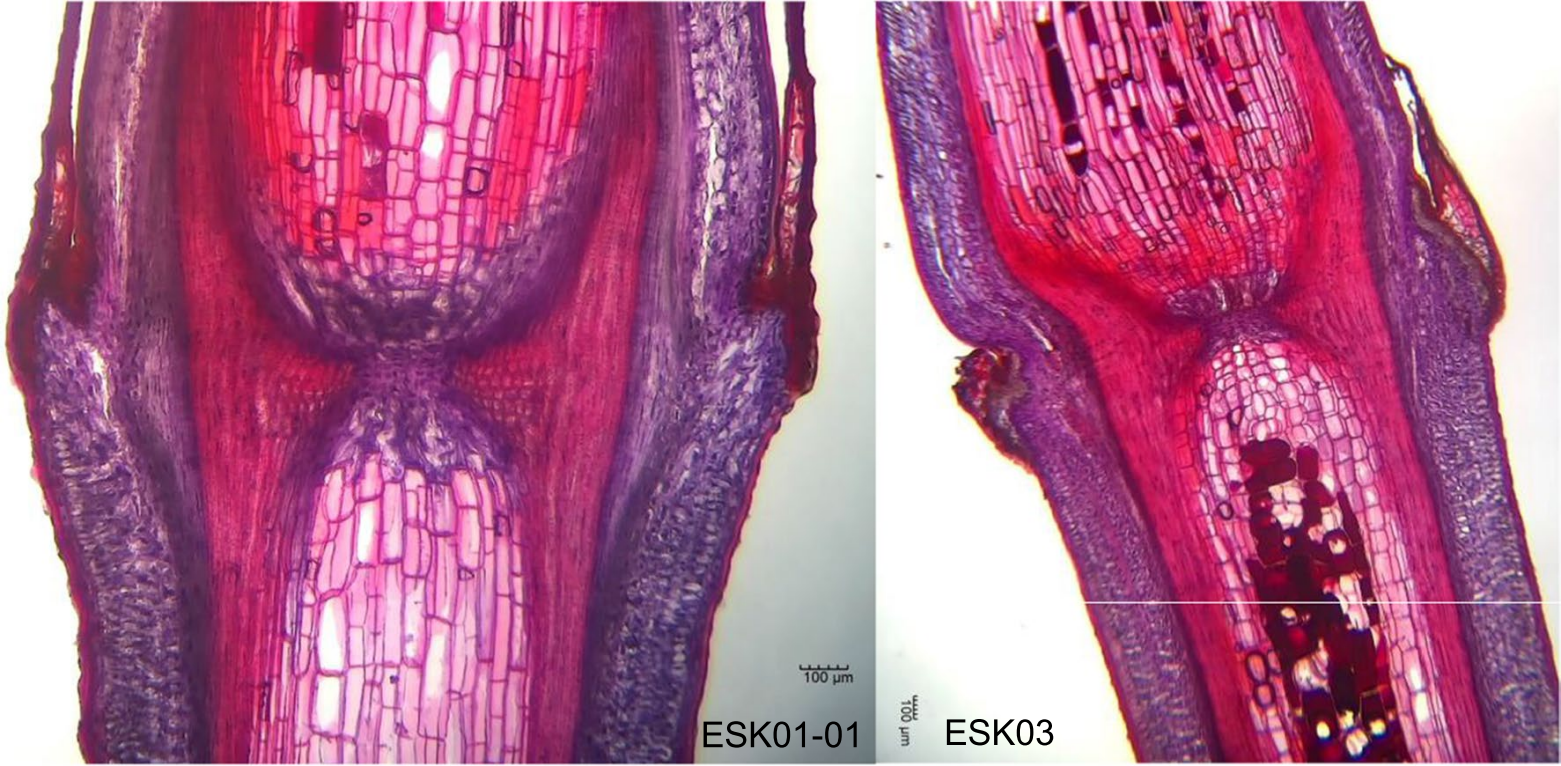
Anatomy of longitudinal slices of ESK01-01 and ESK03. The rooting rates of ESK01-01 and ESK03 were 94.7% and 0.0%, respectively

**Fig. 9:**
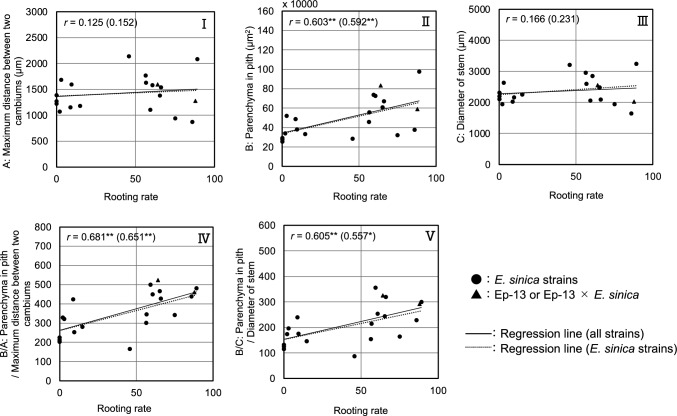
The relationship between rooting rate and measured values at each plant location shown in Fig. 1. **I** The scatter plot of the maximum distance between two cambiums (A) and rooting rate. **II** The scatter plot of parenchyma in pith (B) and rooting rate. **III** The scatter plot of diameter of stem (C) and rooting rate. **IV** The scatter plot of parenchyma in pith/maximum distance between two cambiums (B/A) and rooting rate. **V** The scatter plot of parenchyma in pith/diameter of stem (B/C) and rooting rate. *r* = Pearson’s correlation coefficient. *p < 0.05, **p < 0.01. *n* = 20. The eighteen plots of *Ephedra sinica* are shown in a circle. The two plots of Ep-13 and Ep-13 × *E. sinica* are shown in a triangle. The correlation coefficients out of parentheses were calculated with all of the plots. The correlation coefficients in parentheses were calculated without Ep-13 and Ep-13 × *E. sinica*. The alphabets of A, B, and C on the longitudinal axis legend correspond to the alphabets in Fig. 1

Table 2 lists the measured values at each location shown in Fig. 1 and the rooting, survival, and germination rates of 18 strains and 20 plants. The rooting rates of ESK03, ESS07, and ESS14 were 0.0%. On the other hand, the rooting rate of ESK15-01 was 89.1%, the highest among plants examined. The survival rates of ESK03 and ESS14 were 0.0%. On the other hand, the 98.2% survival rate of ESK15-01 was the highest among *E. sinica* plants. None of the cuttings of ESK01-01, ESK02, ESK03, ESK07, ESK08, ESK12, ESK13, ESS01, ESS07, ESS12, ESS13, ESS14, ESS17 germinated. On the other hand, ESK01-02 had the highest germination rate of 11.3% among *E. sinica* plants. The rooting, survival, and germination rates of Ep-13 were 64.2%, 86.8%, and 32.1%, and those of Ep-13 × *E. sinica* were 88.0%, 100.0%, and 0.0%, respectively. Among *E. sinica* plants, the maximum distance between two cambiums of 872 μm for ESK01-01was the smallest and that of 2141 μm for ESS12 was the largest. The parenchyma in pith of 254,322 μm^2^ for ESK03 was the smallest and that of 975,150 μm^2^ for ESK15-01 was the largest. The stem diameter of 1650 μm for ESK01-01 was the smallest and that of 3251 μm for ESK15-01 was the largest. The parenchyma in pith/maximum distance between two cambiums of 165 for ESS12 was the smallest and that of 500 for ESK15-02 was the largest. The maximum distance between two cambiums, parenchyma in pith, diameter of stem, parenchyma in pith/maximum distance between two cambiums, and parenchyma in pith/diameter of stem of Ep-13 were 1590 μm, 830,712 μm^2^, 2552 μm, 523, and 326, respectively, and those of Ep-13 × *E. sinica* were 1272 μm, 587,403 μm^2^, 2018 μm, 460, and 291, respectively. The representative longitudinal slice from two different strains that have different rooting characteristics is shown in Fig. 8. Table 3 shows the correlation confidences between rooting rate, survival rate, and germination rate and the values of each anatomy. Figure 9 shows scatter plots of rooting rate and the measured values. Significant positive correlations were found between rooting rate, survival rate, and germination rate and parenchyma in pith, parenchyma in pith/maximum distance between two cambiums, and parenchyma in pith/diameter of stem.

**Table 2:** Measured values at each plant location shown in Fig. 1 and rooting rates, survival rates, and germination rates of 18 plants and 20 plants

	Rooting rate (%)	Survival rate (%)	Germination rate (%)	A	B	C	B/A	B/C
				Maximum distance between two cambiums (μm)	Parenchyma in pith (μm^2^)	Diameter of stem (μm)	Parenchyma in pith/maximum distance between two cambiums	Parenchyma in pith/diameter of stem
ESK01-01	86.0	94.7	0.0	872	376,994	1650	438	229
ESK01-02	66.0	75.5	11.3	1537	669,828	2097	428	319
ESK02	65.3	79.4	0.0	1380	607,020	2487	467	244
ESK03	0.0	0.0	0.0	1274	254,322	2196	202	116
ESK07	9.5	22.2	0.0	1598	381,097	2166	253	176
ESK08	2.9	65.2	0.0	1685	521,387	2642	322	197
ESK12	60.6	60.6	0.0	1584	725,901	2861	450	254
ESK13	75.0	90.0	0.0	940	320,146	1943	342	165
ESK15-01	89.1	98.2	5.5	2083	975,150	3251	482	300
ESK15-02	59.3	59.3	11.1	1103	737,004	2067	500	357
ESK16	56.3	89.6	4.2	1766	457,931	2963	301	155
ESS01	8.7	8.7	0.0	1153	487,750	2032	424	240
ESS06	56.7	86.7	5.0	1627	558,854	2604	346	215
ESS07	0.0	1.9	0.0	1387	290,859	2316	211	126
ESS12	45.8	41.7	0.0	2141	283,457	3214	165	88
ESS13	2.0	14.0	0.0	1071	340,122	1951	328	174
ESS14	0.0	0.0	0.0	1226	279,177	2105	225	133
ESS17	14.8	24.1	0.0	1182	332,616	2259	281	147
Ep-13	64.2	86.8	32.1	1590	830,712	2552	523	326
Ep-13 × *E. sinica*	88.0	100.0	0.0	1272	587,403	2018	460	291

Parenchyma in pith/Maximum distance between two cambiums was calculated by dividing parenchyma in pith by Maximum distance between two cambiums

Parenchyma in pith/Diameter of stem was calculated by dividing Parenchyma in pith by Diameter of stem

The alphabets of A, B, and C correspond to the alphabets in Fig. 1

**Table 3:** Correlation between rooting rate, survival rate, and germination rate and each anatomy

	A	B	C	B/A	B/C
	Maximum distance between two cambiums	Parenchyma in pith	Diameter of stem	Parenchyma in pith/maximum distance between two cambiums	Parenchyma in pith/diameter of stem
Rooting rate	0.125 (0.152)	0.603^**^ (0.592^**^)	0.166 (0.231)	0.681^**^ (0.651^**^)	0.605^**^ (0.557^*^)
Survival rate	0.207 (0.231)	0.609^**^ (0.580^*^)	0.236 (0.296)	0.650^**^ (0.601^**^)	0.569^**^ (0.502^*^)
Germination rate	0.175 (0.151)	0.586^**^ (0.612^**^)	0.118 (0.046)	0.499^*^ (0.480^*^)	0.589^**^ (0.717^**^)

Values indicate Pearson’s correlation coefficients

Values not enclosed in parentheses were calculated from all strains

Values in parentheses were calculated without Ep-13 and Ep-13 × *E. sinica*

The alphabets of A, B, and C correspond to the alphabets in Fig. 1

*p < 0.05

**p < 0.01

### Selection of promising strains

#### Quantification of ephedrine and pseudoephedrine

We quantified E and PE in approximately 3,400 *E. sinica* strains cultivated in Shika Town, Hakui-gun, Ishikawa Prefecture from 2015 to 2019. With reference to the Japanese Pharmacopoeia 18th edition (JP18) [23], the strains having no less than 0.7% total alkaloids content (E + PE) were grouped. From the group, 41 strains with various composition ratios of E and PE were selected (Table 4). The E content of the 41 strains ranged from 0.01 to 1.70%; the PE content, from 0.00 to 1.14%; total alkaloids, from 0.76 to 1.76%; and rate of ephedrine, from 0.9 to 100.0%.

**Table 4:** Strains selected on the basis of alkaloid content

	Ephedrine content (%)	Pseudoephedrine content (%)	Total alkaloids content (%)	Rate of ephedrine (%)
ESS18	0.60	0.63	1.23	48.8
ESS19	1.03	0.15	1.18	87.3
ESS20	0.53	0.63	1.16	45.7
ESS21	0.67	0.36	1.03	65.0
ESS22	0.97	0.09	1.06	91.5
ESS23	0.67	0.09	0.76	88.2
ESS24	1.61	0.06	1.67	96.4
ESS25	0.18	1.14	1.32	13.6
ESS26	1.60	0.05	1.65	97.0
ESS27	0.01	1.11	1.12	0.9
ESS28	0.30	0.87	1.17	25.6
ESS29	1.05	0.00	1.05	100.0
ESS30	1.30	0.00	1.30	100.0
ESS31	0.40	0.91	1.31	30.7
ESS32	0.29	1.02	1.31	22.3
ESS33	0.47	1.07	1.54	30.5
ESS34	0.23	0.83	1.06	21.6
ESS35	1.59	0.00	1.59	100.0
ESS36	0.30	0.84	1.13	26.4
ESS37	1.08	0.00	1.08	100.0
ESS38	0.35	0.70	1.06	33.5
ESS39	0.23	0.82	1.04	21.6
ESS40	1.12	0.00	1.12	100.0
ESS41	0.95	0.05	1.00	94.9
ESS42	1.33	0.05	1.38	96.1
ESS43	1.07	0.02	1.09	98.1
ESS44	0.90	0.11	1.01	89.0
ESS45	1.70	0.07	1.76	96.1
ESS46	0.46	0.57	1.03	45.0
ESS47	0.82	0.47	1.29	63.6
ESS48	0.85	0.30	1.15	74.2
ESS49	1.06	0.05	1.11	95.7
ESS50	0.43	0.63	1.05	40.3
ESS51	0.74	0.59	1.33	55.8
ESS52	0.56	0.65	1.21	46.5
ESS53	1.17	0.00	1.17	99.7
ESS54	0.93	0.23	1.16	80.2
ESS55	1.41	0.01	1.42	99.4
ESS56	0.81	0.70	1.51	53.6
ESS57	0.94	0.41	1.36	69.5
ESS58	0.93	0.11	1.03	89.8

The rate of ephedrine was calculated as follows: rate of ephedrine = ephedrine content/(ephedrine content + pseudoephedrine content)

#### Screening by rooting characteristics

**Fig. 10:**
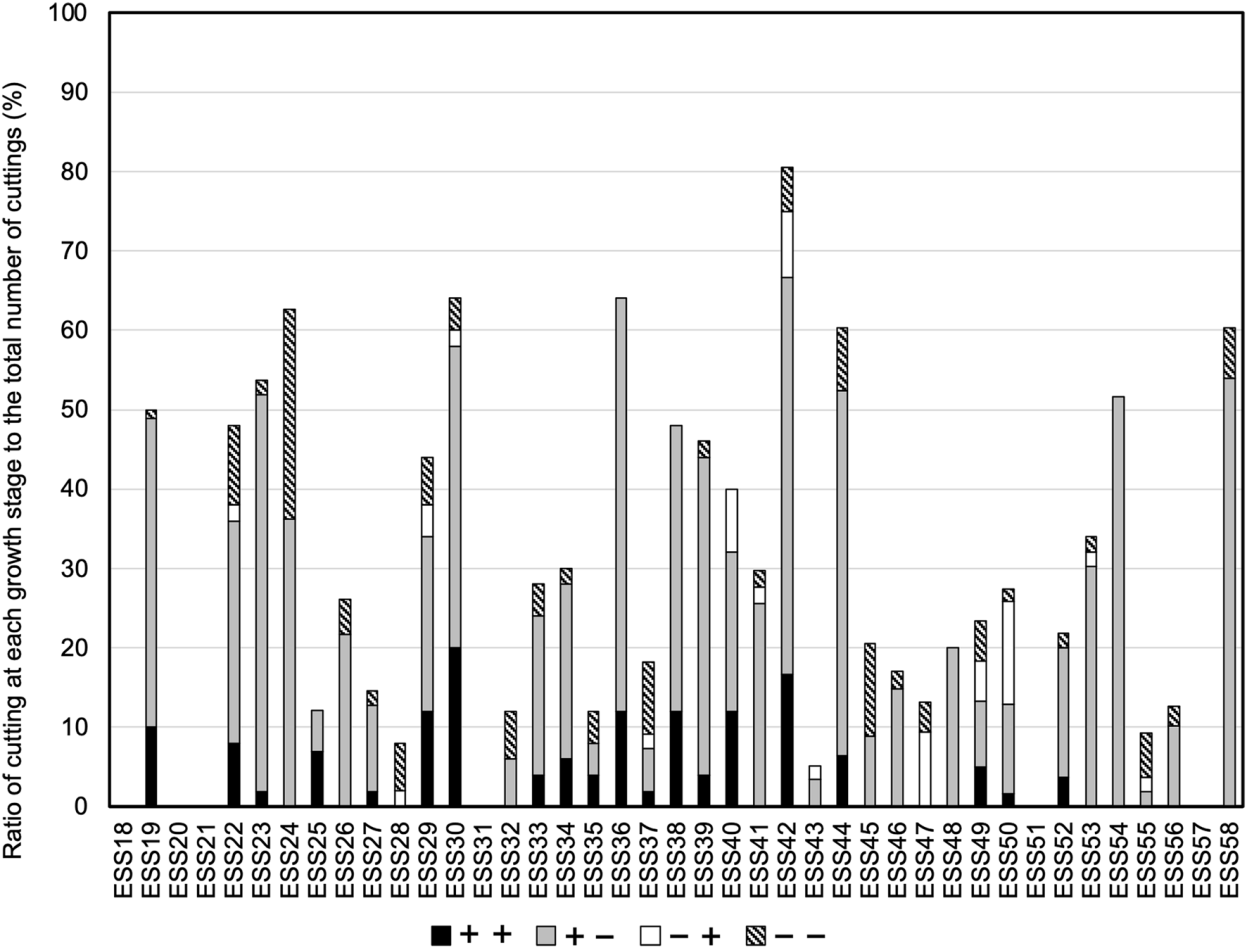
Differences in ratios of cuttings among *E. sinica* strains having characteristic alkaloid contents. +  + : cutting that formed roots and sprouts. + −: cutting that formed roots but not sprouts. −  + : cutting that formed sprouts but not roots. −  − : cutting that survived without withering, although neither roots nor sprouts were formed

Figure 10 shows the ratios of cuttings at various stages for 41 *E. sinica* strains (strain numbers ESS18 to ESS58) cultivated in Shika Town, Hakui-gun, Ishikawa Prefecture in 2020. ESS30 had the highest ratio of +  + cuttings (20.0%), followed by ESS42 (16.7%) and ESS29, 36, 38, and 40 (12.0%). On the other hand, 21 strains had no +  + cuttings. ESS58 had the highest ratio of + − cuttings (54.0%), followed by ESS36 (52.0%) and ESS54 (51.6%); eight strains had no + − cuttings. ESS50 had the highest ratio of −  + cuttings (12.9%), followed by ESS47 (9.4%) and ESS42 (8.3%); 27 strains had no −  + cuttings. ESS24 had the highest ratio of −  − cuttings (26.5%), followed by ESS45 (11.8%) and ESS22 (10.0%); 13 strains had no −  − cuttings.

## Discussion

### Difference in rooting and germination characteristics between strains

The cuttings from 33 *E. sinica* strains were prepared and clarified differences in rooting and germination characteristics in 2019. In the 33 *E. sinica* strains examined, strains with rooting rates of 89.1% and 0.0% were observed, indicating enormous strain differences in rooting characteristics. In addition, there were large differences in the ratio of cuttings that survived without rooting among strains. Such differences in rooting characteristics among races and individuals have been reported in *Cryptomeria japonica* (L.f.) D.Don and *Chamaecyparis obtusa* (Siebold et Zucc.) Endl., and individuals with high rooting rates have been used for breeding [24–26]. Internal factors that affect the rooting of cuttings include the nutritional status of cuttings, rooting inhibitors such as tannins, and differences in anatomy [27]. It has been reported that cuttings prepared from peach cultivars with inferior rooting characteristics have lower carbohydrate content than those prepared from cultivars with good rooting characteristics [28]. Similarly, in *E. sinica* herbal stems, there may be differences in carbohydrate content between strains with low rooting rates and those with high rooting rates. In general, tannin content is low in cuttings prepared from plants that easily root and high in cuttings prepared from plants that do not root [27]. *Vitis kiusiana* Momiy. varieties that are difficult to root have more phenolic compounds in the cuttings than varieties that are easy to root [29]. Similarly, there may be differences in the content of phenolic compounds among *E. sinica* strains. Carbohydrate and phenol contents may function as indicators of rooting characteristics of a strain and should therefore be investigated. Regarding the anatomy, it is said that root primordia exist even before cuttings are prepared in plants that easily root [27]. Therefore, in this study, we investigated the anatomy as well. In addition, plant hormones may also contribute to differences in rooting and germination characteristics, thus meriting future investigations.

### Stability of rooting and germination characteristics

Through investigations of changes in rooting characteristics over a three-year period for the purpose of confirming the stability of rooting characteristics, we found that both ESK01-01 and ESK02 had high rooting and survival rates for three years, and confirmed the stability of the rooting characteristics. In addition, both strains showed higher rooting and survival rates in 2019 than in 2017 and 2018. It is likely that this is due to fertilization conducted in January 2019. It is known that the nutritional status of the parent strain affects the rooting of cuttings [30], and the rooting rate of strains that are cut every year will decrease unless proper fertilization is performed [27]. In the case of ESK01-01, its rooting and survival rates were 59.0% and 89.2% in 2017 but were decreased to 39.5% and 58.0% in 2018. Although this decline might be due to climate effects, we think that one reason is that fertilization was not carried out. Regarding the germination rate, ESK01-01 had 0% germination rate over the three-year period, whereas the germination rate of ESK02 fluctuated from 0 to 18.6% during the same period. The fluctuations in the germination and rooting rates of ESK02 were inconsistent and could be due to the temperature at which mother plants were grown. It has been reported a spate of relatively low temperature days increased the number of germination buds [31]. It is necessary to examine in detail germination characteristics in the rooted cuttings.

### Inheritance of rooting and germination characteristics

Cuttings were made from each of the two parent strains (ESK01-01, ESK15-01) and the new stocks (ESK01-02, ESK15-02) produced by the cuttings for the purpose of confirming the inheritance of rooting characteristics. Cuttings from the mother plants and the new stocks of both strains showed high rooting and survival rates, indicating that the cloned plants produced by cuttings inherited the high rooting rates of the mother plants. As mentioned earlier, although rooting of cuttings may be enhanced by fertilization management, the annual change in rooting characteristics is stable and reflected in the cuttings obtained from the new stocks. In other words, the rooting characteristics of cuttings are one of the important traits in breeding.

### Identification of rooting part by anatomical study

To investigate the internal tissue that affects rooting, longitudinal and transverse sections of the node were prepared and observed, and it was clarified that rooting occurred not only from the outer cortex of the vascular bundle but also from the parenchyma in pith. It has been reported that rooting is enhanced in *Ephedra* plant cuttings prepared by cutting at or immediately below the nodes rather than between the nodes [16]. The difference in rooting rate depending on the cutting position is due to the amount of parenchyma in the cutting position. For cuttings obtained by cutting near the node, adventitious root can also develop from the parenchyma in pith. As mentioned above, the amount of parenchyma on the cut surface is one of the factors that affect rooting. As shown in Fig. 5, a sort of diaphragm of approximately four cells was observed above node. This tissue cut off the pith, left and right xylem, connected to cortex, and merged to the phloem, even before being cut. This diaphragm has already been reported in the previous studies of *E. monostachya* [32] and other *E.* sp. [21]*.* Those relatively small cells were dyed by Delafield's hematoxylin, and cell nucleus were observed, which should be meristem cells. The existence of this tissue is considered the more meristem cells around the node. The existence of this tissue is regarded that the meristem cells around the node were abundant. Those cuttings may have the potential for new tissue development. As shown in Fig. 6, adventitious roots were developed from not only the parenchyma in pith, but also from outer cortex of the vascular bundle as well. By stimulation of cutting, the base of cuttings, especially cortex was hypertrophied, and rooting occurred. The cortex and diaphragm seemed to be merged. This tissue was not observed in *E. sinica*. It may be related to the difficulty of cutting in *E. sinica*. Remarkable staining of dark red in pith was observed, especially in Fig. 5. The tannin-like staining cell in pith was observed in *E. aspera* [33], and some pith cells contained dark brown masses of tannin in *E. gerardiana* [34]. Accordingly, those staining are likely to be tannin.

### Relationship between anatomy and rooting

The relationship between anatomy and rooting rate, survival rate, and germination rate was investigated and uncovered a significant positive correlation between the parenchyma in pith and the rooting rate, survival rate, and germination rate. Parenchyma in pith/maximum distance between two cambiums had the highest positive correlation with rooting rate and survival rate, and parenchyma in pith/diameter of stem had the highest positive correlation with germination rate. In other words, rooting and germination characteristics of cuttings can be estimated by calculating the parenchyma in pith/maximum distance between two cambiums and the parenchyma in pith/diameter of stem. As shown in Fig. 9IV, ESS12, whose rooting rate is 45.8% and parenchyma in pith/Maximum distance between two cambiums is 165, had a different trend from other strains. Eliminating this strain from calculating the correlation coefficient, the value can be reached 0.757. The strain that shows the different trends, is possible to be affected greatly by other factors like hormones.

It has been reported that *E. sinica* individuals show large differences in anatomy and their herbal stem diameters range from 1.11 to 2.44 mm[35]. In this study as well, herbal stem diameter measured approximately 1.65 to 3.25 mm and varied markedly among strains, although it showed no correlation with the rooting rate, survival rate, or germination rate. On the other hand, the parenchyma in pith showed a positive correlation with the rooting rate, the survival rate, and the germination rate. Furthermore, the correlation coefficient increased between rooting rate and parenchyma in pith/maximum distance between two cambiums or herbal stem. As we have shown, the parenchyma in pith is where adventitious roots develop, so it is expected to have a positive correlation with the rooting rate. In addition, the parenchyma in pith had a positive correlation with the survival rate, the reason being that not only adventitious roots but also callus and new xylem developed from the parenchyma. In strains with abundant parenchyma in pith, we surmised that the cut surface was sealed by the development of callus, thereby protecting the cut surface and preventing nutrient outflow. In addition, it is expected that the development of new xylem will result in water absorption and survival even though adventitious roots do not grow.

### Selection of promising strains

#### Quantification of ephedrine and pseudoephedrine

The ratio of E to PE is genetically controlled as described in the Introduction [2]. Furthermore, clone seedlings can inherit the alkaloid accumulation characteristics of the mother plants [11, 12]. The pollen mating methods for *Ephedra* plants were invented [35] and used them to generate an enormous number of *E. sinica* resources that include not only strains that have high alkaloid content but also those that have low alkaloid content. It has reported that Ephedra herb induced FcεRI internalization in mast cells, thereby inhibiting antigen-induced IgE-dependent degranulation [36]. However, it is likely that the effect was not caused by alkaloid because Ephedra herb that had low alkaloid content produced the same effect as Ephedra herb that had high alkaloid content. In other words, we surmise that FcεRI internalization induced by Ephedra herb is attributable not to E but to other active components [36]. Moreover, alkaloid-free Ephedra has been invented as EFE (ephedrine alkaloid-free Ephedra herb extract) [37–41]. Considering the side effects of E, strains that have low alkaloid content are important resources as well. We also keep *E. sinica* strains that rarely contain alkaloid. However, it is defined in JP18 that Ephedra herb should have no less than 0.7% total alkaloids content (E + PE). Therefore, in this study, the strains with characteristic alkaloid content were selected. Those strains are important resources for breeding new *E. sinica* cultivars.

Zhang et al. [42] recommended that E-rich Ephedra herb should be used for Kampo formulations such as Maoto (麻黄湯) and Makyokansekito (麻杏甘石湯), which are used to induce perspiration and as an antitussive. On the other hand, it is also recommended that PE-rich Ephedra herb should be used for Kampo formulations such as Bofutsushosan (防風通聖散) [42], which is used as an anti-inflammatory and analgesic, and for weight loss.

#### Screening by rooting characteristics

To screen promising strains, the strains with high or characteristic alkaloid content were selected and used to prepare cuttings. ESS19, ESS23, ESS30, ESS36, ESS38, ESS42, ESS44, ESS54, and ESS58, which have approximately 50% or higher rooting rates, were suitable for use as cuttings. On the other hand, ESS30 and ESS42 showed 22.0% or higher germination rates and were suitable for the cutting breeding method. There was no correlation between rooting characteristics or germination characteristics and E content, PE content, and total alkaloids content. Several compounds have been reported as candidates for Ephedra herb bioactive compound, such as volatile oils, phenolic acids, and high molecular mass condensed tannin [38, 43]. In future research, we intend to select more promising strains from the strains that were selected in this study, taking into consideration other compounds as well.

## Conclusion

It is expected to produce clone seedlings to ensure uniform quality of herbal medicine and establish breeding methods for *Ephedra* plants. In this study, to improve the efficiency of *Ephedra* cuttings, an efficient method for the evaluation of rooting characteristics was investigated and selected promising strains. Examination of differences in rooting and germination characteristics between strains revealed that the rooting and germination characteristics varied among strains. Approximately 90% of cuttings prepared from some strains formed roots, whereas cuttings from other strains failed to form roots. The stability and inheritance of rooting characteristics were also investigated and found that rooting characteristics were stable for several years and inherited by new stocks. To evaluate the rooting and germination characteristics without preparing cuttings, the correlation between those characteristics and anatomy was determined, that is, we estimated rooting and germination characteristics on the basis of anatomical observations. Furthermore, we selected promising strains on the premise of not only rooting and germination characteristics but also alkaloid content. The selected strains are useful as breeding resources of *Ephedra* plants. In conclusion, we have invented an efficient method for the evaluation of rooting and germination characteristics, which has led to the selection of promising strains of *E. sinica*.

## Data Availability

The datasets generated during and/or analysed during the current study are available from the corresponding author on reasonable request.
