# Correction: Evaluation of rooting characteristics of *Ephedra* cuttings by anatomy and promising strain selection based on rooting characteristics and alkaloid content

**DOI:** 10.1007/s11418-024-01807-0

**Published:** 2024-04-03

**Authors:** Yoshitomi Kudo, Hirokazu Ando, Ai Kaneda, Honoka Ito, Kazuki Umemoto, Si-ran Ni, Masayuki Mikage, Yohei Sasaki

**Affiliations:** 1 Laboratory of Molecular Pharmacognosy, Graduate School of Medical Sciences, Kanazawa University, Kakuma-Machi, Kanazawa, Ishikawa 920-1192 Japan; 2 Laboratory of Medicinal Plant Resources, Department of Bio-Resource Development, Faculty of Agriculture, Tokyo University of Agriculture, 1737 Funako, Atsugi, Kanagawa 243-0034 Japan

**Correction: Journal of Natural Medicines (2023) 77:327-342** 10.1007/s11418-023-01680-3

The article Evaluation of rooting characteristics of Ephedra cuttings by anatomy and promising strain selection based on rooting characteristics and alkaloid content, written by Yoshitomi Kudo, Hirokazu Ando, Ai Kaneda, Honoka Ito, Kazuki Umemoto, Si ran Ni, Masayuki Mikage, Yohei Sasaki, was originally published Online First without Open Access. After publication in volume 77, issue 2, page 327–342 the author decided to opt for Open Choice and to make the article an Open Access publication. Therefore, the copyright of the article has been changed to © The Author(s) 2024 and the article is forthwith distributed under the terms of the Creative Commons Attribution 4.0 International License, which permits use, sharing, adaptation, distribution and reproduction in any medium or format, as long as you give appropriate credit to the original author(s) and the source, provide a link to the Creative Commons license, and indicate if changes were made. The images or other third party material in this article are included in the article’s Creative Commons licence, unless indicated otherwise in a credit line to the material. If material is not included in the article’s Creative Commons licence and your intended use is not permitted by statutory regulation or exceeds the permitted use, you will need to obtain permission directly from the copyright holder. To view a copy of this licence, visit http://creativecommons.org/licenses/by/4.0.

The original article was updated.

